# Platelet-Activating Factor-Induced Inflammation in Obesity: A Two-Sided Coin of Protection and Risk

**DOI:** 10.3390/cells14070471

**Published:** 2025-03-21

**Authors:** Smaragdi Antonopoulou

**Affiliations:** Department of Nutrition and Dietetics, School of Health Sciences and Education, Harokopio University, 17671 Athens, Greece; antonop@hua.gr

**Keywords:** adipocytes, macrophages, LpPLA_2_, PAF metabolism, uncoupling protein-1, PPARγ, ether lipids, high-fat diet, Mediterranean diet

## Abstract

Obesity, marked by excessive fat accumulation, especially abdominal, is a global health concern with significant public impact. While obesity-associated chronic unresolved inflammation contributes to metabolic dysfunctions, acute inflammation supports healthy adipose tissue remodeling and expansion. Platelet-activating factor (PAF), a “primitive” signaling molecule, is among the key mediators involved in the acute phase of inflammation and in various pathophysiological processes. This article explores the role of PAF in fat accumulation and obesity by reviewing experimental data from cell cultures, animals, and humans. It proposes an emerging biochemical mechanism in an attempt to explain its dual role in the healthy and obese adipose tissue, including also data on PAF’s potential involvement in epigenetic mechanisms that may be linked to the “obesity memory”. Finally, it highlights the potential of natural PAF modulators in promoting functional adipose tissue, thermogenesis, and obesity prevention through a healthy lifestyle, including a Mediterranean diet rich in PAF weak agonists/PAF receptor antagonists and regular exercise, which help maintain controlled PAF levels. Conversely, in cases of obesity-related systemic inflammation with excessive PAF levels, potent PAF inhibitors like ginkgolide B and rupatadine may help mitigate metabolic dysfunctions with PAFR antagonists potentially enhancing their effects synergistically.

## 1. Introduction

In recent decades, the high prevalence of obesity has emerged as a major concern, affecting not only high-income countries but also some middle-income nations. Between 1990 and 2022, the global proportion of children and adolescents up to 19 years old living with obesity surged from 2% to 8%, while the percentage of adults over 18 with obesity more than doubled, reaching 16% [1].

Obesity is a condition that poses significant health risks and has consequently been the subject of extensive study across various scientific fields. While obesity-associated chronic unresolved inflammation is considered a contributing mechanism to metabolic dysfunctions such as insulin resistance, the acute phase of inflammation seems to be important for healthy adipose tissue remodeling and expansion through release of angiogenic factors, endogenous resolution mechanisms including pro-inflammatory mediators, as well as by expression of an active form of inhibitor of kB. Studies, particularly those involving transgenic mice, suggest that pro-inflammatory mediators increase energy expenditure and lipolysis rates [2,3,4,5,6].

**Figure 1:**
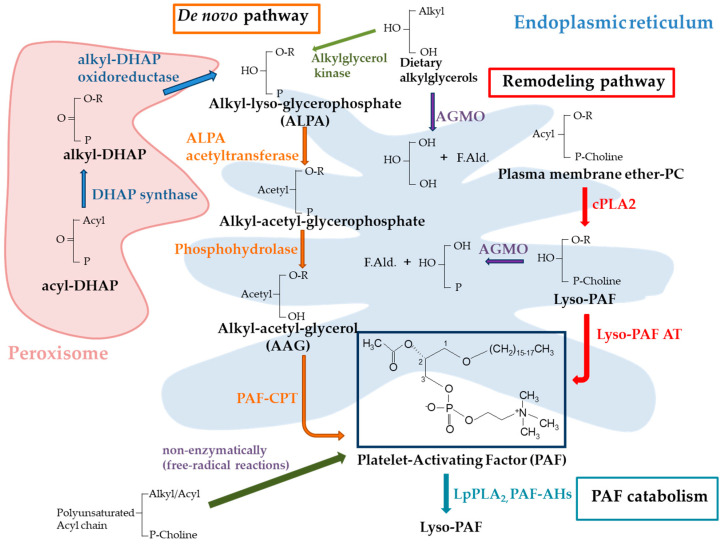
A schematic presentation of PAF metabolic pathways. The red arrows and red-labeled enzymes depict the remodeling pathway, while the orange arrows and orange-labeled enzymes represent the de novo pathway. The light blue arrow and enzymes represent the PAF catabolic pathways. Blue arrows and enzymes illustrate the reactions occurring in the peroxisome. The dark green arrow signifies the non-enzymatic formation of PAF, whereas the dark blue arrows indicate potential substrates for AGMO. Lastly, the green arrow shows the conversion of dietary alkylglycerols to ALPA. AGMO: alkylglycerol monooxygenase; ALPA: alkyl-lyso-glycerophosphate; cPLA2: cytoplasmic phospholipase A2; DHAP: dihydroxyacetonephosphate; F.Ald.: fatty aldehyde; LpPLA_2_: lipoprotein-associated phospholipase A_2_; Lyso-PAF AT: acetyl-CoA:lyso–platelet-activating factor acetyltransferase; PAF-AHs: PAF-specific acetylhydrolases; PAF-CPT: 1-alkyl-2-acetyl-sn-glycerol cholinephosphotransferase; PC: phosphatidylcholine.

Among the pro-inflammatory mediators that are implicated in the acute phase of inflammation is platelet-activating factor (PAF, 1-O-alkyl-2-acetyl-*sn*-glycero-3-phosphocholine) [7], a “primitive” signaling molecule existing since the earliest stages of life on earth and found in a wide variety of organisms, including pro- and eukaryotic cells, plants, animals, and humans [8]. PAF is produced by various cells, including endothelial cells, platelets, macrophages, monocytes, neutrophils, and mast cells, in response to different stimuli, where it exerts pleiotropic effects [9]. The levels of PAF in cells, tissues, and biological fluids are primarily regulated through two biosynthetic pathways, the de novo and remodeling pathways, as well as the catabolic pathways. The de novo pathway is responsible for the constitutive production of PAF and contributes to the physiologic levels of PAF needed for normal cellular function, with the key enzyme, 1-alkyl-2-acetyl-*sn*-glycerol cholinephosphotransferase (PAF-CPT), catalyzing the synthesis of PAF from 1-O-alkyl-2-acetyl-glycerol by adding phosphocholine [10] (Figure 1). The remodeling pathway begins with cytoplasmic phospholipase A2 (cPLA2) acting on existing membrane ether-linked choline-containing phospholipids, leading to the formation of lyso-PAF that is subsequently acetylated by acetyl-CoA:lyso–platelet-activating factor acetyltransferases (Lyso-PAF AT) to produce PAF. Two isoforms of Lyso-PAF AT are known; one is activated during inflammation (LPCAT2), while the other is calcium-independent and appears not to be involved in inflammatory processes [11]. In addition, PAF can be formed non-enzymatically during uncontrolled free-radical reactions that also generate “PAF-like activity lipids” (PLAL) such as oxidized phospholipids [12]. PAF catabolism is performed by intracellular PAF-specific acetylhydrolases (PAF-AHs) and its plasma isoform, lipoprotein-associated phospholipase A_2_ (Lp-PLA_2_) [13].

It should be noted that the formed PAF may either remain within the cell, functioning as an intracellular mediator, or be displayed on the cell membrane for juxtracrine signaling, or be released for paracrine action. Although the circumstances under which biosynthesized PAF will act through any of the above mechanisms are not fully clarified, it appears to be cell type-dependent. While monocytes release a significant amount of PAF, endothelial cells, on the other hand, express it on their cell membrane, and finally, leukocytes may release PAF following an appropriate trigger. Additionally, PAF levels, under normal conditions, are maintained at low concentrations by de novo synthesis while during an inflammatory response its biosynthesis peaks through the remodeling pathway. PAF exerts its biological effects by binding to its G-protein coupled receptor (PAFR), or by activating the inflammasome [14]. PAF-R is widely expressed across various cells and tissues, including immune cells, platelets, epithelial, and endothelial cells, and in multiple organs such as the spleen, kidney, liver, lung, heart, intestine, and brain [15]. PAFR that is located on the plasma and nuclear membrane couples with different types of G-proteins such as Gαo, Gαi, Gβγ, and Gαq, subsequently activating several signal transduction pathways in a cell- and tissue-dependent manner [16]. In many instances, PAF as well as PLAL binding to PAFR leads to a pro-inflammatory phenotype, while activation of the PAFR in dendritic cells and during the phagocytosis of apoptotic cells by macrophages is linked to a suppressive phenotype [17,18].

PAF participates in several physiological processes including angiogenesis, reproduction, apoptosis, wound healing, modulation of blood pressure, neuronal function, and brain development while inappropriate/excess of PAF signaling leads to pathological conditions associated with inflammation and cellular damage and death such as allergies, asthma, cancer, renal diseases, infections, and cardiovascular diseases [9,19,20]. Regarding reproduction, it has been demonstrated that embryos of all mammalian species produce and release PAF that acts as a dual survival/trophic factor [21]. A recent article also documented that in infants, adipose tissue macrophages (ATMs) biosynthesize PAF from precursor molecules present in breast milk, which functions in an autocrine manner to promote the differentiation of adipocytes into beige fat by increasing uncoupling protein-1 (UCP1) transcription through the interleukin-6/Janus kinase/signal transducer and activator of transcription 3 (IL-6/JAK/STAT3) signaling pathway [22].

This review aims to update and discuss the experimental data on the role of PAF in fat accumulation and obesity, drawing from studies involving cell cultures, animals, and humans. Additionally, it proposes a potential biochemical mechanism to explain PAF’s dual role in healthy and obese adipose tissue. A literature search was conducted in the PubMed and Scopus databases up to January 2025, using combinations of terms related to platelet-activating factor (PAF), including ether lipids, PLA2, PAF receptor (PAF-R), and lipoprotein-associated phospholipase A_2_ (Lp-PLA_2_), alongside terms related to obesity, adipose tissue, adipocytes, adiposity, diet, weight, glucose, insulin, lipolysis, triglycerides, and fatty acids.

## 2. Animal Studies and In Vitro Experiments

In the animal studies, PAFR-knockout (PAFR-KO) mice have been exclusively used as experimental models to demonstrate that PAFR deficiency leads to increased adiposity and weight gain, either with aging or nutrient overload, accompanied by a pro-inflammatory macrophage phenotype. Research data from mice demonstrated that PAFR is expressed in adipose tissue (AT), primarily in brown adipose tissue (BAT) but also in white adipose tissue (WAT). Specifically, PAFR presence in WAT has been established in the mature adipocytes and stromal vascular cells (SVCs) in epididymal WAT. PAFR deficiency, even in the absence of nutrient overload, led to an obese state characterized by increased body weight, visceral fat, and enhanced lipogenesis (Table 1). The results revealed that PAF or PAFR signaling increase energy expenditure and may play a role in regulating UCP1 expression in BAT as well as in beige adipocytes located within WAT through PAF-induced β3-adrenergic receptor expression [23]. Under a high-carbohydrate diet, PAFR-KO mice, despite the absence of insulin resistance, presented decreased resistin levels and increased levels of leptin and peroxisome proliferator-activated receptor gamma (PPARγ), a key regulator of genes that promote fat storage in AT. Notably, a protective effect against diet-induced adipose inflammation was observed, as adipocytes from these animal models failed to secrete tumor necrosis factor-α (TNF-α) or IL-6 under hyperglycemic conditions [24]. In response to a high-fat diet (HFD), impaired glucose tolerance was observed in PAFR-KO mice, while serum leptin levels and the mRNA expression of all lipases, including fatty acid synthase, remained unchanged. PAFR deficiency appears to disrupt the phosphatidylinositol-3-kinase (PI3K)/Akt (also known as protein kinase B) pathway by reducing Akt phosphorylation, leading to impaired regulation of glucose and lipid metabolism [25]. HFD feeding did not seem to affect PAF levels. Additionally, a HFD induced fatty liver in both PAFR-KO and wild-type (WT) mice. The mRNA levels of inflammatory cytokines in the liver and epididymal WAT were not different between PAFR-KO and WT mice, implying that the PAF signal may not significantly affect AT inflammation in vivo [26].

ATMs remained unaffected in terms of both the number and phenotype (M1 and M2) in young PAFR-KO mice while the macrophage infiltration of WAT, including M1 macrophage-specific protein CD11c within crown-like structures in AT, increased with age and also under HFD, contributing to a pro-inflammatory phenotype of ATMs. Notably, PAFR-KO mice fed the HFD did not present any improvement in TNF-α expression levels and in the infiltration of CD11c-positive macrophages suggesting that the PAF signal does not significantly contribute to the accumulation of pro-inflammatory macrophages in the AT; however, on the other hand, PAF promotes macrophage differentiation into classically activated phenotypes [23,24,25,26]. Macrophage accumulation in AT is observed during its expansion, probably driven by adipocyte necrosis, tissue remodeling, or micro-hypoxia, but it is also recorded in mice during fasting and early weight loss, implying a role of lipolysis in ATM recruitment [29]. While ATMs are the primary contributors to the inflammatory profile, other cells of innate immunity—such as eosinophils, neutrophils, lymphocytes, dendritic cells, and mast cells—also play key roles in AT inflammation by either attracting macrophages or affecting their phenotype. A 24 h fasting period in mice has been associated with increased neutrophil recruitment into AT, accompanied by elevated levels of pro-inflammatory cytokines within the tissue. Notably, when PAFR-KO mice were subjected to the same fasting period, significantly reduced loss of fat and epididymal AT was recorded, along with a markedly smaller increase in pro-inflammatory cytokines compared to WT mice. This evidence indicates that PAF signaling co-regulates cytokine production in non-hematopoietic cells, including adipocytes, which plays a crucial role in driving early fat-mass reduction [27].

The reduced fat accumulation and lower expression of adipogenic genes as well as the up-regulation of UCP1 expression, have also been reported in 3T3-L1 adipocytes under the treatment of PAF [26,28]. When mouse 3T3-L1 fibroblasts were differentiated in a PAF-containing medium, they showed reduced gene expression of PPARγ and CCAAT/enhancer-binding protein-α (C/EBP-α), which are key in adipocyte differentiation. While some of this effect may stem from resident AT cells, the potential role of PAF in this differentiation process cannot be ruled out. This is supported by data showing that both PAF catabolic enzymes, PAF-AH (isoform Ib) and Lp-PLA_2_, negatively regulate canonical Wnt signaling, suggesting that PAF signaling plays a role in β-catenin translocation and activation of downstream Wnt genes, consequently ensuring the survival of committed preadipocytes and inhibiting adipogenesis [28,30,31,32].

PAF metabolic enzymes are present in both the WAT and BAT of mice and humans. Specifically, PAF can be synthesized via the de novo pathway in mouse and human adipocytes, as well as in human ATMs, while the remodeling pathway contributes to PAF production in both adipocytes and ATMs of mice and humans. Regarding PAF catabolism, PAF-AH is localized in adipocytes, whereas Lp-PLA_2_ activity is detected in ATMs [22,23,26]. Most studies to date have focused on PAF biosynthesis in ATMs through the remodeling pathway, which is primarily activated by inflammatory stimuli and significantly contributes to PAF levels. This emphasis is largely due to the presence of alkylglycerol monooxygenase (AGMO) in both adipocytes and ATMs, as its activity reduces precursor compounds for the de novo PAF biosynthetic pathway while also decreasing lyso-PAF levels—the key precursor for the remodeling pathway [22,33,34]. However, recent findings suggest an alternative perspective. AGMO knockdown during 3T3-L1 adipocyte differentiation led to a significant accumulation of vinyl ether-phospholipids (plasmalogens), yet no substantial changes were observed in the relative abundances of 1-O-alkyl-lyso-phospholipids, the substrates of Lyso-PAF AT [35]. Similarly, altering AGMO activity had no impact on PAF or lyso-PAF levels during murine macrophage differentiation [36]. Additionally, lipidomic analyses have detected ether-linked phospholipids within the lipid droplet monolayer of AT [37]. From a methodological perspective, the hormones included in the 3T3-L1 adipocyte differentiation medium have been shown to inhibit either the production or action of PAF. In other words, the standard protocol prevents the clarification of the role of endogenous biosynthesized PAF [38,39,40].

## 3. Human Studies

Although limited, the data from human studies align with the above findings. The most striking result came from the Comprehensive Assessment of Long-term Effects of Reducing Intake of Energy (CALERIE) clinical trial demonstrating that the expression of the gene Pla2g7 encoding Lp-PLA_2_ was inhibited in the abdominal subcutaneous AT of healthy humans undergoing moderate calorie restriction for 2 years and was accompanied with improved thymopoiesis and increased mitochondrial bioenergetics including fatty acid oxidation and anti-inflammatory responses (Table 2). Their results were also confirmed in Pla2g7 knockout mice where favorably altered macrophage phenotypes that promote tissue remodeling as well as increased eosinophils were detected along with increased AT lipolysis [41]. A prior study on obese women also found that a calorie-restricted diet, which significantly reduced obesity-related anthropometric indices and improved lipid profiles, led to a notable decline in plasma Lp-PLA_2_ levels [42]. It has also been suggested that increased total energy intake may induce Lp-PLA_2_ activity [43]. Knowing that Lp-PLA_2_ is the key plasma enzyme responsible for PAF degradation, these results suggest a potential beneficial role of PAF in adipose tissue. The positive link between Lp-PLA_2_ and fat accumulation has been previously observed in healthy adults and in obese adolescents, with and without type 2 diabetes [44,45]. In addition, waist circumference and waist-to-hip ratio showed a negative correlation with PAF levels. Notably, men, who tend to store fat viscerally and exhibit significantly higher waist circumference and waist-to-hip ratios, had lower PAF levels—particularly PAF bound to cellular structures—and higher activity of both catabolic enzymes and Lyso-PAF AT compared to women [44,46]. Furthermore, Lp-PLA_2_ activity was positively associated with upper-body and total adiposity in healthy men, suggesting a compensatory role in response to adiposity-related changes [47]. PAFR mRNA expression in omental WAT was irreversible associated with body weight, body mass index (BMI), and body fat mass in severely obese subjects. In these subjects, PAFR mRNA expression was more pronounced in SVF than adipocytes [28]. Of note, an autocrine cycle of new PAF synthesis and PAFR activation has been established [48]. Lastly, in studies involving monozygotic twins, ether-phospholipid concentrations were notably lower in the obese co-twins. Their concentrations showed a negative correlation with measures of subcutaneous obesity and a positive correlation with insulin sensitivity [49]. The above results indicate the beneficial effect of PAF levels in AT on obesity indices.

All studies suggest that the reduction in Lp-PLA_2_ leads to a non-obese phenotype, highlighting the need for careful interpretation of the results. Blood macrophages have been identified as the primary source of this enzyme, with their differentiation state playing a crucial role in determining its baseline levels and responsiveness to microenvironmental stimuli. Its secretion is regulated by various factors, including inflammatory and non-inflammatory substrates, cytokines, and steroid hormones [50]. It is also known that Lp-PLA_2_ action not only lowers PAF levels but also reduces oxidized phospholipids (PLAL) and at the same time, it promotes the production of lyso-phosphatidylcholine (LPC). The production of LPC, which has been shown to be involved in inflammation and endothelial dysfunction, along with epidemiological data indicating increased Lp-PLA_2_ levels in cardiovascular diseases, has led to the characterization of this enzyme as exerting pro-inflammatory properties. The activation of proliferator-activated receptor alpha (PPARα)—a key regulator of fatty acid oxidation and triacylglycerol (TAG) reduction in the liver—has been shown to significantly increase the expression of Lp-PLA_2_ and elevate LPC levels in the liver, while also appearing to minimize PAF signaling by reducing PAFR expression in mature macrophages [51]. Moreover, Lp-PLA_2_, through the production of LPC, activates PPARα [50]. Additionally, PAF induces Lp-PLA_2_ expression in monocyte/macrophages, suggesting a regulatory mechanism in response to increased PAF levels [52]. These findings support the idea that Lp-PLA_2_’s role in hepatic fat accumulation is determined by the balance between the concentrations of its substrate (PAF) and its product (LPC). On the other hand, regarding PPARγ, which is predominantly expressed in AT, the aforementioned findings from experimental animals and adipocytes indicate that PAF reduces the gene expression of PPARγ, leading to reduced adipogenesis in AT, while PPARγ inhibition down-regulates Lp-PLA_2_ mRNA and protein expression [50]. These seemingly conflicting findings can be reconciled by considering macrophage origins. Many resident tissue macrophages arise during early embryonic development and persist in adulthood without relying on circulating monocytes. However, during inflammation, blood-derived macrophages infiltrate tissues, replacing embryonically established macrophages. Notably, the origins, maintenance, and polarization of ATMs—even the conventional M1/M2 phenotypes—remain poorly understood [53].

## 4. Discussion

**Figure 2:**
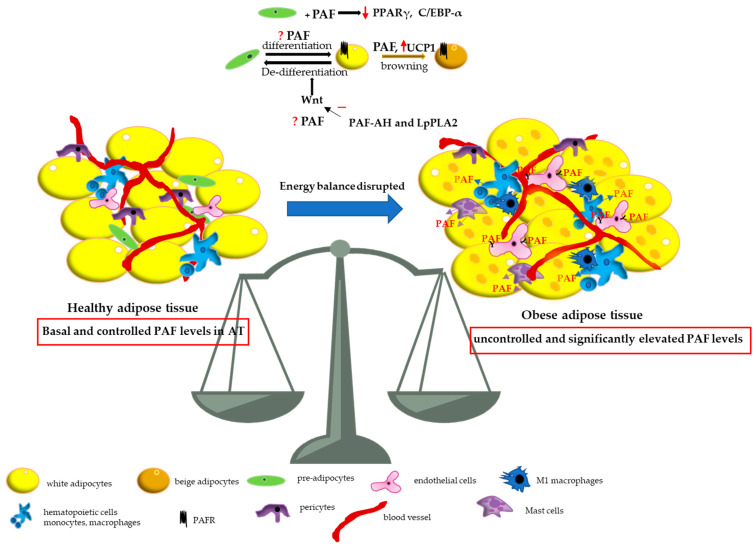
A simplified schematic representation of the role of PAF in adipose tissue. A healthy adipose tissue (AT) is characterized by basal and controlled PAF levels while an obese adipose tissue displays uncontrolled and significantly elevated PAF levels. ↑: refers to increase; ↓: refers to decrease.

Collectively, the above data support a beneficial role of PAF production, under non-chronic inflammatory circumstances such as through the de novo pathway in adipocytes and ATMs. PAF seems to function in an autocrine manner and maintains beige and brown adipocytes thus regulating energy expenditure by thermogenesis (Figure 2). Recent studies have demonstrated that BAT is active and functional in adult humans although the CALERIE participants did not show any change in UCP1 expression in AT [41,54]. Based on the results from cells and experimental animals, PAF does not seem to significantly contribute to AT lipolysis. Regarding the role of PAF in circulating TAG levels, there are limited and conflicting results, with some pointing to its contribution to an increase in lipoprotein lipase activity and hydrolysis of TAG when PAF levels are low, while others suggest that it may lead to elevated serum TAG along with peripheral lipolysis [55,56]. Even though PAF’s involvement in cell differentiation processes has been established [57,58], and some data indicate PAF’s implication in adipocyte differentiation, the results are still rare and inconclusive. A healthy, lean AT contains anti-inflammatory immune cells (e.g., eosinophils, M2 macrophages, Th2 cells) that support AT normal function and promote tissue regeneration and homeostasis. Whether, under these conditions of healthy AT, PAF participates with an immunosuppressive role either in the phagocytosis of dead adipocytes by M2 macrophages or in the maintenance of the anti-inflammatory microenvironment, as seen in other cases [17,18], has not yet been studied. When the energy balance equation is disrupted either by overnutrition or reduced energy expenditure, adipogenesis is increased to store excess calories along with the formation of new adipocytes. As a result, visceral WAT expands, leading to hypertrophic tissue characterized by larger adipocyte size. This is associated with the accumulation of blood monocytes/macrophages in AT, infiltration of B and T cells, immune cell activation, hypoxia, fibrosis, increased adipocyte death, and oxidative stress. However, the precise sequence in which these processes occur remains unclear. In obese AT, monocytes differentiate to pro-inflammatory macrophages (M1), other pro-inflammatory immune cells are also increased (e.g., neutrophils, mast cells, Th1 cells) resulting in inflammation and adipose dysfunction [59]. Although PAF signaling does not seem to play a significant role in the accumulation of M1 macrophages in AT, the onset of systemic inflammation, oxidative stress, and local hypoxia in hypertrophic AT triggers extensive PAF production, primarily via the remodeling pathway along with the generation of PLAL. This increased PAF production, combined with enhanced PAFR signaling, contributes to the amplification of inflammation and to the induction of a fibrotic response [9,26,60,61,62]. It has also been demonstrated that under inflammatory stress, PAF promotes lipid accumulation in human mesangial cells by dysregulating LDL receptor function [63]. Notably, kidney endothelial cells are the most susceptible to obesity and exhibited the least recovery following a weight-loss diet [64]. Conversely, insulin resistance has been shown to inhibit PAF biosynthesis in the kidneys of rats, likely leading to reduced plasma PAF levels [65].

An abundance of evidence from recent years has demonstrated that obesity extends beyond merely consuming excess calories or experiencing an energy imbalance; it is also influenced by a variety of epigenetic mechanisms that play a crucial role in the development of diet-induced obesity [66]. Very recently, in an article by Hinte et al., it was presented that in both subcutaneous and visceral fat, AT cells retain epigenetic modifications even two years after significant weight loss. These lasting epigenetic changes are associated with reduced metabolism and impaired adipocyte function, including decreased activation of the Akt signaling pathway, reduced phospholipid biosynthesis, and lower mitogen-activated protein kinase phosphatase-1 activity. Additionally, these modifications promote the activation of pathways leading to fibrosis and apoptosis [67]. In addition, diet-induced obesity also leads to the lasting epigenetic reprogramming of the innate immune system [68]. The relationship between PAF metabolism and the observed epigenetic reprogramming may be bidirectional. On the one hand, these changes could influence PAF levels and dysregulate—probably up-regulate—its signal [69], while on the other hand, PAF itself may contribute to these modifications. It has been shown that PAF reduced the expression of DNA methyltransferases DNMT1 and DNMT3b, while increasing the expression of the histone acetyltransferase p300 and promoting histone H3 acetylation [70]. Additionally, PAF has been associated with the induction of p21 expression, a key regulator of the cell cycle, through epigenetic mechanisms [71]. In asthma, where PAF has a clear role, PAF appears to influence miRNAs that regulate inflammation such as miR-146a [72].

Unhealthy dietary patterns, including hypercaloric and high-fat diets, have been shown to trigger unfavorable epigenetic changes. Conversely, physical activity and the intake of bioactive nutritional compounds can promote beneficial epigenetic modifications. An increasingly diverse array of nutrients with epigenetic influence encompasses minerals, vitamins, polyphenols, and a wide range of plant-derived compounds. Among the phytochemicals reported to influence body weight and energy expenditure through epigenetic mechanisms are resveratrol from grapes, garlic-derived compounds like diallyl trisulfide and diallyl disulfide, organosulfur compounds such as sulforaphane found in cruciferous vegetables, and the tea catechin epigallocatechin-3-gallate [66]. It is of particular interest that almost all the aforementioned phytochemical compounds have been shown to inhibit PAF biosynthesis and/or action [73,74,75,76,77]. Additionally, the biosynthetic enzyme of the remodeling pathway showed a negative association with healthy dietary patterns, specifically those rich in whole-wheat products and olive oil, as well as those emphasizing fruits, nuts, and herbal drinks [78]. Adopting a healthy lifestyle, including balanced dietary patterns such as the Mediterranean diet—which contains PAF weak agonists, lipid compounds that act through PAFR at higher concentrations, in other words PAFR antagonists—may promote functional and healthy AT since mild inflammation with moderately elevated but controlled PAF levels localized in AT promotes thermogenesis and contributes to obesity prevention [79,80]. Regarding olive oil that is the main source of the fatty components of the Mediterranean diet, recent data have shown that a sustained increase in olive oil consumption is linked to a decrease in body weight among middle-aged adults [81]. Additionally, it has been shown that exercise reduces liver PAF levels in mice fed a HFD by affecting its metabolic enzymes [82]. Conversely, in individuals with obesity, where chronic inflammation driven by hematopoietic cells leads to uncontrolled and significantly elevated PAF levels, the use of potent PAF inhibitors such as ginkgolide B (BN52021), a selective PAF receptor antagonist and rupatadine (RUPAFIN), a dual-action antihistamine and PAF inhibitor, could help mitigate metabolic dysfunctions [83,84,85]. In the latest case, the use of PAFR antagonists may work synergistically with potent PAF inhibitors to suppress chronic inflammation triggered by PAF [86].

## 5. Conclusions

PAF appears to play a dual role in regulating body weight and, consequently, obesity. Physiologically controlled levels of PAF in AT and its microenvironment seem to exert a beneficial autocrine effect, contributing to the maintenance of healthy AT. On the other hand, in conditions of oxidative stress imbalance and inflammation, elevated levels of PAF are produced by blood cells and the endothelium, which through paracrine action and juxtracrine signaling, lead to inflammation and thrombosis. It appears, therefore, that PAF levels determine its beneficial or harmful role in organs and tissues, in a manner similar to that of reactive oxygen species or cytokines.

## Figures and Tables

**Table 1:** Studies investigating the PAFR deficiency in animal models.

Experimental Model	Study Design	Main Findings	Ref.
PAFR-KO and WT male mice	mice were fed CD for up to 36 w single injection of the β3-AR agonist, CL-316,243 (25 μg/kg)	PAFR-KO vs. WT ↑ body weight gain ↑ epididymal WAD at 12 w ↑ liver weight at 36 w ↑ TNFα, IL-6, CCL3 mRNA expression in the epididymal WAT at > 24 w ↓ UCP1 and UCP3 mRNA in the BAT and UCP2 mRNA in the WAT ↓ thermogenesis ↑ LPCAT2 and cPLA2α mRNAs in BAT	[23]
PAFR-KO and WT male mice	mice were fed a (i) CD or (ii) HCD for 8 w	PAFR-KO HCD vs. WT HCD ↑ body weight ↑ VAI ↑ lipogenesis ↑ PPARγ ↓ resistin ↑ leptin ↓ protein levels of TNFα, IL-6, IL-1β, IL-10, CCL3, and CCL5 ↓ HSL mRNA expression in AT	[24]
PAFR-KO and WT BALB/c mice	mice were fed a (i) CD or (ii) HFD for 12 w	PAFR-KO vs. WT ↑ IL12/IL10 in epididymal ATM ↑ insulin resistance PAFR-KO HFD vs. WT HFD ↑ body weight gain ↑ epididymal fat ↑ liver weight ↑ insulin resistance	[25]
PAFR-KO and WT male mice	mice were fed a (i) CD or (ii) HFD for 12 w	PAFR highly expressed in adipocytes and SVC of epididymal WAT PAFR weakly expressed in preadipocytes cPLA2a and LPCAT2 highly expressed in BAT and WAT PAFR-KO HFD vs. WT HFD ↑ body weight gain ↑ epididymal WAT ↑ TNFα mRNA ↑ CD11c-positive macrophages into epididymal WAT ↑ fasting serum glucose	[26]
PAFR-KO and WT BALB/c mice	mice were fed a HCD and subjected to 24 h fasting	PAFR-KO fasting vs. WT fasting ↓ decrease in epididymal AT ↓ IL-6, TNFα, IL-1β, IL-10, and TGF-β in AT	[27]
PAFR-KO and WT C57BL/6 male mice	mice subjected to BM transplantation and fed a CD or HCD for 8 w (i) PAFR-KO -BM→PAFR-KO; (ii) WT-BM→WT (iii) PAFR-KO-BM→WT, and (iv) WT-BM → PAFR-KO	PAFR-KO-BM → PAFR-KO HCD vs. WT-BM → WT HCD ↓ O_2_ consumption PAFR-KO-BM→PAFR-KO HCD, WT-BM→PAFR-KO HCD vs. WT-BM → WT HCD ↑ leptin ↓ TNFα, IL-6 WT-BM → PAFR-KO CD vs. WT-BM → WT CD ↑ serum glucose ↑ insulin PAFR-KO-BM → PAFR-KO, WT-BM→PAFR-KO vs. WT-BM → WT ↓ rolling of leukocytes in AT	[28]

AR: adrenergic receptor; AT: adipose tissue; ATM: adipose tissue macrophages; BAT: brown adipose tissue; BM: bone marrow; CCL3: chemokine(C-C motif) ligand 3; CCL5: chemokine(C-C motif) ligand 5; CD: chow diet; cPLA2α: cytosolic phospholipase A2α; HCD: high-refined carbohydrate-containing diet; HFD: high-fat diet; HSL: hormone-sensitive lipase; IL-1β: interleukin-1β; IL-6: interleukin-6; IL-10: interleukin-10; IL-12: interleukin-12; LPCAT2: acyl-CoA:lysophosphatidylcholine acyltransferase 2; PAFR: PAF receptor; PAFR-KO: PAF receptor knockout; PPARγ: proliferator-activated receptor gamma; SVC: stromal vascular cells; TGF-β: transforming growth factor β; TNFα: tumor necrosis factor α; UCP1,2,3: uncoupling protein-1, 2, and 3; VAI: visceral adiposity index; WAD: white adipose deposits; WAT: white adipose tissue; WT: wild-type. ↑: refers to increase; ↓: refers to decrease.

**Table 2:** Studies investigating the role of PAF in human subjects.

Study Population	Study Intervention	Measurements	Main Findings	Ref.
43 obese subjects (both sexes, BMI 46.7 ± 6.9 kg/m^2^)	-	anthropometric data Gly, Ins, HOMA-IR, TC, TAG, HDL- and LDL-chol, Apo A1 and B IL-6, TNFα, IL-10, adip: omental AT gene expression	PAFR expression in omental WAT negatively correlated with BW, BMI, and FM	[28]
28 obese women (BMI 38.0 ± 4.9 kg/m^2^)	low-calorie diet for 16 w	anthropometric data Gly, Ins, HOMA-IR, TC, TAG, HDL- and LDL-chol, Apo A1 and B Lp-PLA_2_ activity LDL phenotype	↓ Lp-PLA_2_ activity at 16 w changes in Lp-PLA_2_ activity were correlated only with the changes in VLDL-chol	[42]
52 men and 48 age- and BMI-matched women	-	anthropometric data DXA measurements Gly, TC, TAG, HDL- and LDL-chol, CRP lyso-PAF-AT, PAF-CPT, PAF-AH activities in leukocytes and Lp-PLA_2_ in plasma Blood PAF levels	Lp-PLA_2_ activity positively correlated with TAG, TC, LDL-chol in men and women PAF-AH in leukocytes positively correlated with CRP in men and women upper and total adiposity measures positively associated with Lp-PLA_2_ activity in men WC and W/H negatively correlated with PAF levels	[44,47]
14 MZ twin pairs, (8 male and 6 female pairs) one co-twin with BMI 25 kg/m^2^ the other with BMI 30 kg/m^2^	-	DXA measurements, MRI Gly, Ins, Ins sensitivity, TC, TAG, HDL- and LDL-chol, leptin, adip, CRP Lipidomics’ analysis	obese vs. non-obese co-twins ↑ LPCs ↓ ether PL ether PL negatively correlated with subcutaneous obesity and positively with insulin sensitivity	[49]
65 obese with T2D and 72 obese subjects without T2D	-	anthropometric data TC, HDL- and LDL-chol, HbA1c, leptin, adip Lp-PLA_2_ activity	obese with T2D vs. obese without T2D ↓ Lp-PLA_2_ activity Lp-PLA_2_ activity positively associated with BMI and negatively associated with leptin and adip	[45]
284 subjects (both sexes)	-	anthropometric data Gly, Ins, TC, TAG, HDL- and LDL-chol, UA, creatinine, SGOT, SGPT, γ-GT, IL-6, adip Lp-PLA_2_ activity	Lp-PLA_2_ activity negatively correlated with HDL-chol, SGOT, SGPT, and adip total energy intake positively associated with Lp-PLA_2_ activity	[43]
238 healthy subjects	(i) 2y CR by about 14% (ii) ad libitum control group	anthropometric data TDEE, AT gene expression thymic function, fat composition of thymus, sjTRECs	2y CR ↑thymic mass ↓FM ↑ mitochondrial biogenesis, PPAR-α–FAO ↓ expression of the gene encoding Lp-PLA_2_ in AT	[41]
100 subjects with high and low risk of CVD	-	anthropometric data TC, LDL-, HDL- and non-HDL-chol, TAG, Lp-PLA_2_ activity	Lp-PLA_2_ positively correlated with LDL- and non-HDL-chol medium positive correlation between Lp-PLA_2_ and absolute CVD risk score	[46]

Adip: adiponectin; Apo A1: apolipoprotein A1; Apo B: apolipoprotein B; AT: adipose tissue; BMI: body mass index; BW: body weight; CR: calorie restriction; CRP: c-reactive protein; CVD: cardiovascular disease; DXA: dual-energy x-ray absorptiometry; FAO: fatty acid oxidation; FM: fat mass; Gly: glycose; γ-GT: γ-glutamyltransferase; HDL-chol: high density lipoprotein cholesterol; HOMA-IR: homeostatic model assessment for insulin resistance; Ins: insulin; IL-6: interleukin-6; IL-10: interleukin-10; LDL-chol: low-density lipoprotein cholesterol; LPCs: lysophosphatidylcholines; Lp-PLA_2_: lipoprotein-associated phospholipase A_2_; lyso-PAF-AT: acetyl-CoA:lyso–platelet-activating factor acetyltransferase; MRI: magnetic resonance imaging; MZ: monozygotic; PAF-AH: PAF acetyhydrolase; PAF-CPT: 1-alkyl-2-acetyl-sn-glycerol cholinephosphotransferase; PAFR: PAF receptor; PL: phospholipids; PPARα: proliferator-activated receptor alpha; SGOT: glutamic oxaloacetictransaminase; SGPT: glutamic pyruvic transaminase; sjTRECs: signal joint T-cell receptor excision circles; TAG: triglycerides; TC: total cholesterol; TDEE: total daily energy expenditure; TNFα: tumor necrosis factor α; UA: uric acid; VLDL-chol: very low-density lipoprotein cholesterol; WAT: white adipose tissue; WC: waist circumference; W/H: waist-to-hip ratio. ↑: refers to increase; ↓: refers to decrease.

## Data Availability

No new data were created or analyzed in this study.

## Abbreviations

The following abbreviations are used in this manuscript:

AGMO	alkylglycerol monooxygenase
Akt	protein kinase B
AT	adipose tissue
ATMs	adipose tissue macrophages
BAT	brown adipose tissue
BMI	body mass index
C/EBP-α	CCAAT/enhancer-binding protein-α
cPLA2	cytoplasmic phospholipase A2
HFD	high-fat diet
IL-6	interleukin-6
JAK	Janus kinase
LPC	lyso-phosphatidylcholine
LpPLA_2_	lipoprotein-associated phospholipase A_2_
Lyso-PAF AT	acetyl-CoA:lyso–platelet-activating factor acetyltransferases
PAF	platelet-activating factor
PAF-AHs	PAF-specific acetylhydrolases
PAF-CPT	1-alkyl-2-acetyl-*sn*-glycerol cholinephosphotransferase
PAFR	PAF G-protein coupled receptor
PAFR-KO	PAFR-knockout
PI3K	phosphatidylinositol-3-kinase
PLAL	PAF-like activity lipids
PPARα	proliferator-activated receptor alpha
PPARγ	proliferator-activated receptor gamma
STAT3	Signal transducer and activator of transcription 3
SVC	stromal vascular cells
TAG	triacylglycerols
TNF-α	tumor necrosis factor-α
UCP1	uncoupling protein-1
WAT	white adipose tissue
WT	wild-type
